# Removal of Deformity of Rickety Limbs

**Published:** 1890-12-27

**Authors:** 


### REMOVAL OF DEFORMITY OF RICKETY LIMBS.

Dr. Max Schüller treats the curvature of the femur in rickety children by forcible straightening of the bone. The child is put under chloroform and a long strong nickel-plated steel apparatus, with aseptic padding is applied to the limb, which is then forcibly straightened by the mechanism, and retained in position for from five to ten days. There is naturally some pain on recovery from anæsthesia, but he has never met with fever or inflammation.

